# Infusion-related reactions and premedication patterns in ublituximab-treated multiple sclerosis patients: a multicenter real-world study

**DOI:** 10.3389/fneur.2026.1840196

**Published:** 2026-07-16

**Authors:** Anas Z. Nourelden, Parker R. Scott, Sam I. Hooshmand, Izabela Mazur, Felicia Mada, Tesiley Ash, Nidhi Patel, Jacob Rube, Kalyan Yarraguntla, Carey Deluca, Patti Yager-Stone, Ryan Havens, Mitchell Wallin, Anza B. Memon

**Affiliations:** 1 Department of Neurology, Wayne State University, School of Medicine, Detroit, MI, United States; 2 Department of Neurology, Medical College of Wisconsin, Milwaukee, WI, United States; 3 John D. Dingell VA Medical Center, Detroit, MI, United States; 4 Department of Neurology, Detroit Medical Center, Detroit, MI, United States; 5 Washington VA Medical Center, Washington, DC, United States; 6 Portland VA Medical Center, Portland, OR, United States

**Keywords:** B-cell, infusion reactions, multiple sclerosis, premedication optimization, ublituximab

## Abstract

**Background:**

Ublituximab is a glycoengineered monoclonal antibody that effectively reduces multiple sclerosis (MS) relapses but carries a significant risk of infusion-related reactions (IRRs), which is relatively higher than that reported for some other anti-CD20 agents. This study aims to characterize real-world IRRs associated with ublituximab and describe premedication practices across participating centers.

**Methods:**

This multicenter retrospective study included ublituximab-treated MS patients identified from existing medical records (a convenience sample) at MS centers across the United States. Data on demographics, MS phenotype, premedication regimens, and IRRs were collected. Variables were summarized, and Fisher’s exact test was used to compare the occurrence and timing of IRRs across baseline and premedication categories. Also, associations between premedication strategies and IRRs were assessed by logistic regression. We conducted statistical analyses using JAMOVI (v2.5). *p*-values were adjusted for multiple comparisons.

**Results:**

Among 89 patients with multiple sclerosis (PwMS) treated with ublituximab, 34.8% (*n* = 31) experienced IRRs. All patients received premedication, with the acetaminophen dose being the only component significantly associated with overall IRR occurrence (*p* = 0.006). Significant differences in reaction timing (immediate versus delayed) were observed across certain corticosteroid and antihistamine categories. All IRRs were mild to moderate in severity.

**Conclusion:**

Approximately one-third (34.8%) of ublituximab-treated patients experienced IRRs, with varied timing and symptom patterns. No independent predictors of IRRs were identified. Importantly, all IRRs observed in this study were mild to moderate, suggesting that ublituximab infusions were generally well tolerated in this real-world cohort.

## 1 Introduction

Multiple sclerosis (MS) is a chronic inflammatory and neurodegenerative disorder of the central nervous system (CNS) (1, 2). The disease involves an immune-mediated attack on the myelin sheath surrounding nerve fibers, causing plaque formation and disrupting nerve conduction (2). Widespread neurodegeneration also occurs, contributing to progressive disability accumulation (1, 2).

Several disease-modifying therapies (DMTs) are currently approved for the treatment of MS, targeting immune pathways to reduce relapse rates and delay progression. Among B-cell depleting agents, ublituximab (marketed as Briumvi®) is a novel glycoengineered anti-CD20 monoclonal antibody approved for clinically isolated syndrome (CIS), relapsing–remitting MS (RRMS), and active secondary progressive MS (ASPMS) (3–6). Phase 3 ULTIMATE I/II trials demonstrated significant reductions in annualized relapse rates (0.08) and gadolinium-enhancing lesions (0.33) with ublituximab compared with teriflunomide (0.18–0.19, 0.78, respectively) (7, 8). These clinical benefits led to regulatory approval in December 2022 (5). Glycoengineering distinguishes ublituximab from other anti-CD20 antibodies (e.g., rituximab, ofatumumab, ocrelizumab), potentially offering higher efficacy and tolerability (9, 10). Its engineered Fc region enhances FcγRIIIa affinity, increasing antibody-dependent cellular cytotoxicity (ADCC) (8, 11). This allows lower doses, shorter infusion times, and potentially improved safety (8, 11).

Despite its efficacy in reducing relapse rates, ublituximab was associated with frequent infusion reactions, leading to infusion discontinuation in some instances. In Phase 3 ULTIMATE I/II trials, pooled infusion-related reaction (IRR) incidence with ublituximab was 47.7% (44.0% in ULTIMATE I; 51.5% in ULTIMATE II) versus 12.2% for teriflunomide. Most IRRs were mild to moderate, with Grade ≥3 events in 2.8% of patients; two Grade 4 IRRs occurred (one anaphylaxis at the second infusion and one transient lymphopenia). Conversely, the Phase 2 trial reported 50% IRR incidence (all Grade 1–2), highest at first infusion (44%), with no adverse events (AE)-related discontinuations and 94% completing the study (11). IRRs peaked at the first infusion (43.3%), declining to <10% thereafter, with 69.5% of patients experiencing no recurrence (7).

During the ULTIMATE trials, premedication (antihistamine and glucocorticoid ± acetaminophen) was administered, and IRR management also included infusion rate reduction (7). Since premedication protocols in clinical practice often differ from the standardized regimens used in clinical trials, there remains limited real-world data regarding how premedication practices vary across centers and how IRRs present outside controlled trial settings.

Therefore, this study aimed to analyze multicenter real-world data to characterize IRRs associated with ublituximab and to explore potential relationships between premedication practices and reported IRRs.

## 2 Methods

### 2.1 Study design

This is a multicenter, retrospective study that included ublituximab-treated MS patients identified from existing medical records (convenience sample) from five participating MS centers across the United States over the years 2023–2025, following Institutional Review Board (IRB) approval obtained from the two primary contributing sites, which provided the majority of patient data: the Veterans Affairs MS centers (IRB HCR-HP-00043983-10) and the Medical College of Wisconsin (IRB PRO00013874). All procedures were conducted in accordance with institutional and federal regulations, including the Declaration of Helsinki. Patient privacy and confidentiality were strictly maintained, and no identifiable personal information was used. Given the retrospective design, the requirement for written informed consent was waived by the respective IRBs.

### 2.2 Criteria and data collection

We included patients diagnosed with MS (any subtype) who received at least one ublituximab infusion (administered per standard protocol: first infusion over 4 h, starting at 10 mL/h and gradually increased to 100 mL/h; subsequent infusions over 1 h, starting at 100 mL/h and increased to 400 mL/h), with available follow-up data after infusion to identify any IRRs. We extracted data from electronic health records for all eligible patients. Baseline demographic data included age, sex, and race. Moreover, the clinical characteristics included the MS phenotype (CIS, RRMS, or SPMS), disease duration, and the prior history of DMT. Details of ublituximab administration were recorded on all infusions within the healthcare systems. We collected comprehensive information on premedication regimens, including the type, dose, and route of administration for all antihistamines, corticosteroids, and antipyretics. Premedication strategies were administered as part of routine clinical care, allowing for an observational analysis of varied risk-mitigation patterns and their associated IRR outcomes.

### 2.3 Endpoints and definitions

The primary endpoint was the occurrence of IRRs. An IRR was defined as any adverse sign or symptom occurring after initiating the infusion and could be attributed to the drug. We categorized IRRs based on their timing of onset (12, 13): Immediate IRR: Occurring within hours of the infusion. Early Post-Infusion IRR: Occurring hours to days after the infusion. Late Post-Infusion IRR: Characterized by fatigue and malaise occurring weeks after the infusion. For analysis, early post-infusion IRRs (hours to days) and late post-infusion IRRs were grouped together and referred to as ‘delayed IRRs’. This grouping is used for analytical purposes only and does not imply mechanistic equivalence. To minimize reporting bias, all IRRs were identified and graded by independent reviewers at each site using CTCAE v5.0 criteria. Premedication protocols were not standardized across centers, reflecting real practice.

### 2.4 Statistical analysis

Descriptive analyses were conducted to summarize all characteristics. The distribution of continuous variables was assessed for normality using the Shapiro–Wilk test. Continuous variables were presented as mean and standard deviation (SD), or median and interquartile range (IQR). Categorical data were summarized as frequencies and percentages (%). To analyze differences in IRR occurrence and timing across baseline and different premedication categories, we used the Chi-square test or Fisher’s exact test selected for small sample sizes and expected low cell counts in the contingency tables. The Mann–Whitney U test was used to compare the age between patients with and without IRRs. A multivariable binomial logistic regression model was constructed to evaluate the independent predictive value of premedication strategies on the occurrence of an IRR. The dichotomous outcome variable was the presence or absence of an IRR. The primary predictor variable was the premedication strategy. The model was adjusted for a set of *a priori*-defined potential confounders: MS type, DMT-naïve status, sex, and race. The results of the regression analysis are presented as Odds Ratios (ORs), with corresponding 95% confidence intervals (CIs) and *p*-values. All statistical analyses were performed using JAMOVI (Version 2.5), a user-friendly software built on the R statistical environment (14). Missing data were handled using available-case analysis; for each variable, only patients with complete data for that variable were included in the corresponding analysis, while the remaining patients contributed to all analyses for which their data were complete. The threshold for statistical significance was set at an alpha level of *p* < 0.05 for all two-tailed tests. To account for multiple comparisons, false discovery rate (FDR) correction was applied to the univariate analyses using the Benjamini-Hochberg method. Consistent with the exploratory nature of the study, an FDR threshold of q < 0.10 was prespecified to identify associations warranting further investigation. Both unadjusted *p*-values and FDR-adjusted q-values are reported.

## 3 Results

### 3.1 Cohort characteristics

A total of 89 patients were included in the analysis. Most patients had RRMS (*n* = 76, 85.4%). The mean age of the cohort was 43.9 ± 11.9 years (range, 18–75). The study population included 54 females (60.7%) and 35 males (39.3%). The ethnic distribution was 62.5% White (*n* = 55), 36.4% Black (*n* = 32), and 1.1% Asian (*n* = 1). Sixty-eight patients (76.4%) had previously received DMT. The most common prior DMTs were interferon beta-1a (*n* = 22, 24.7%), fumarates (*n* = 21, 23.6%), ocrelizumab (*n* = 21, 23.6%), glatiramer acetate (*n* = 19, 21.3%), and ofatumumab (*n* = 14, 15.7%). Fifty-one patients (57.3%) were anti-CD20 naïve. The baseline demographic and clinical characteristics are detailed in Table 1.

**Table 1:** Baseline characteristics of the enrolled patients.

Characteristics	Overall (*N* = 89)
MS type	
RRMS	76 (85.4%)
SPMS	13 (14.6%)
Age	
Mean (SD)	43.9 (11.9)
Range	18.0–75.0
Sex	
Female	54 (60.7%)
Male	35 (39.3%)
Race	
Black	32 (36.4%)
White	55 (62.5%)
Asian	1 (1.1%)
DMT Naïve	
No	68 (76.4%)
Yes	21 (23.6%)
DMT type	
Interferon beta-1a	22 (24.7%)
Rituximab	7 (7.9%)
Fumarates	21 (23.6%)
Ocrelizumab	21 (23.6%)
Ofatumumab	14 (15.7%)
Glatiramer acetate	19 (21.3%)
Others [one or more] (Alemtuzumab, Fingolimod, Ozanimod, Betaseron, Teriflunomide)	30 (33.7%)
Anti-CD20 Naïve	
No	38 (42.7%)
Yes	51 (57.3%)

### 3.2 Incidence and nature of infusion-related reactions (IRRs)

Thirty-one patients (34.8%) experienced an IRR. The most frequently reported symptoms were fatigue and malaise, which were often presented as delayed IRRs. Among the immediate IRRs, throat-related complaints (itching, tightness, or tingling) and flushing were the most frequent, whereas rigors and back pain occurred less commonly. First-dose reactions occurred in 15 patients (of those with available data), whereas second-dose reactions were less frequent (*n* = 5). These findings are presented in Table 2.

**Table 2:** Infusion-related reactions (IRR) characteristics.

Infusion-related reaction parameters	Total (All = 89 or IRR = 31)
Infusion related reactions (IRR)	
No	58 (65.2%)
Yes	31 (34.8%)
Type of Infusion related reactions (IRR)	
Delayed IRR	16 (51.6%)
Immediate IRR	14 (45.2%)
Immediate and delayed IRR	1 (3.2%)
First dose reaction	
N-Miss	13
No	3 (16.7%)
Yes	15 (83.3%)
Second dose reaction	
N-Miss	13
No	13 (72.2%)
Yes	5 (27.8%)
Immediate IRR (within hours)	
No	16 (51.6%)
Yes	15 (48.4%)
Early post-infusion IRR (hours to days)	
No	19 (61.3%)
Yes	12 (38.7%)
Late post-infusion IRR (fatigue and malaise, weeks)	
No	23 (74.2%)
Yes	8 (25.8%)
Symptoms of IRR	
Fatigue	9 (29%)
Malaise	8 (25.8%)
Throat symptoms (itching, tingling, tightness, or difficulty swallowing)	7 (22.6%)
Nausea	6 (19.4%)
Headache	4 (12.9%)
Respiratory symptoms (chest tightness, dyspnea, pressure, heaviness)	4 (12.9%)
Rigors (chills)	3 (9.7%)
Flushing	3 (9.7%)
Back pain	2 (6.5%)
Muscle stiffness/spasms	2 (6.5%)
Ear itching	2 (6.5%)
Anxiety, off balance, tingling/numbness, upper abdominal pressure, upper extremity pain, bloating/gassy/stomach irritation, increased heart rate, cold, vomiting, diarrhea, flu-like symptoms.	1 (3.2%) each

### 3.3 Premedication strategies

All patients received premedication: a combination of an antihistamine, a corticosteroid, and an antipyretic. The majority of patients received second-generation antihistamine (*n* = 49, 55.1%), followed by first-generation antihistamines (*n* = 36, 40.4%), and a small proportion received both (*n* = 4, 4.5%). The most frequently used corticosteroid class was intermediate-acting (*n* = 66, 74.2%), with methylprednisolone 100 mg IV (*n* = 59, 66.3%) being the most common specific agent. Acetaminophen 650 mg PO (*n* = 74, 83.1%) was the most used antipyretic. A detailed breakdown of all premedication components and strategies is available in Table 3.

**Table 3:** Premedication strategies: detailed characteristics.

Premedications	Overall (*N* = 89)
Antihistamine generation	
First and second generations	4 (4.5%)
First-generation	36 (40.4%)
Second-generation	49 (55.1%)
Antihistamine (type, dose, and route)	
Diphenhydramine 50 mg IV	25 (28.1%)
Cetirizine 10 mg PO	48 (53.9%)
Diphenhydramine 25 mg PO	8 (9.0%)
Diphenhydramine 50 mg IV + loratadine 10 mg PO	4 (4.5%)
Loratadine 10 mg PO	1 (1.1%)
Diphenhydramine 25 mg IV	3 (3.4%)
Corticosteroid acting class	
Short acting	17 (19.1%)
Short and intermediate acting	6 (6.7%)
Intermediate acting	66 (74.2%)
Corticosteroid (type, dose, and route)	
Hydrocortisone 100 mg IV	17 (19.1%)
Hydrocortisone 100 mg IV + Prednisone 40 mg PO	1 (1.1%)
Methylprednisolone 100 mg IV	59 (66.3%)
Methylprednisolone 125 mg IV	5 (5.6%)
Hydrocortisone 100 mg IV + Prednisone 5 mg PO	5 (5.6%)
Methylprednisolone 62.5 mg IV	2 (2.2%)
Antipyretic (type, dose, and route)	
Acetaminophen 650 mg PO	74 (83.1%)
Acetaminophen 100 mg PO	2 (2.2%)
Acetaminophen 1 g PO	13 (14.6%)
Premedication strategies (detailed)	
First-generation antihistamine + short-acting corticosteroid + acetaminophen	16 (18.0%)
First-generation antihistamine + short and intermediate acting corticosteroid + acetaminophen	2 (2.2%)
First-generation antihistamine + intermediate-acting corticosteroid + acetaminophen	18 (20.2%)
Second-generation antihistamine + intermediate-acting corticosteroid + acetaminophen	48 (53.9%)
First and second generations antihistamine + short-acting corticosteroid + acetaminophen	1 (1.1%)
Second-generation antihistamine + short and intermediate acting corticosteroid + acetaminophen	1 (1.1%)
First and second generations antihistamine + short and intermediate acting corticosteroid + acetaminophen	3 (3.4%)

### 3.4 Factors associated with infusion-related reactions

In the univariate analysis (Table 4), there was no statistically significant association between the occurrence of an IRR and baseline characteristics, including MS phenotype (*p* = > 0.99), age (*p* = 0.172), sex (*p* =0.931), or race (*p* = 0.878). CD20 naïve status was not significantly associated with IRR occurrence *p* = 0.371. No significant associations were observed between IRR incidence and most premedication components, including antihistamine type or class (*p* = 0.24, 0.22), corticosteroid type or class *p* = 0.48, 0.33, or overall premedication strategy (*p* = 0.22). Acetaminophen (*p* = 0.006) was the only significant factor. An association between acetaminophen dosing categories and IRR occurrence was observed among anti-CD20 naïve patients. However, no significant association was observed in patients who had previously been treated with anti-CD20 agents, Supplementary Table 1. Across acetaminophen dosing categories, variation in IRR frequency was apparent. However, meaningful inferences are precluded by the uneven distribution of patients among subgroups, with some dose categories containing insufficient numbers for robust comparison.

**Table 4:** Difference between different categories of baseline and premedication in terms of infusion-related reactions (IRR) [Yes Vs. No reaction].

Dependent: infusion related reactions (IRR)	Categories	No 58 (65.2)	Yes 31 (34.8)	Total 89	*p* value	*q* value
MS type	RRMS	49 (84.5)	27 (87.1)	76 (85.4)	1.000	> 0.99
	SPMS	9 (15.5)	4 (12.9)	13 (14.6)		
Age	Median (IQR)	46.5 (39.2 to 51.0)	41.0 (31.5 to 52.0)	45.0 (35.0 to 51.0)	0.172	0.561
Sex	Female	35 (60.3)	19 (61.3)	54 (60.7)	0.931	1.000
	Male	23 (39.7)	12 (38.7)	35 (39.3)		
Race	Black	22 (37.9)	10 (33.3)	32 (36.4)	0.878	1.000
	White	35 (60.3)	20 (66.7)	55 (62.5)		
	Asian	1 (1.7)	0 (0.0)	1 (1.1)		
DMT Naïve	No	46 (79.3)	22 (71.0)	68 (76.4)	0.377	0.566
	Yes	12 (20.7)	9 (29.0)	21 (23.6)		
	Anti-CD20 Naïve	No	27 (46.6)	11 (35.5)			38 (42.7)	0.371	0.561
		Yes	31 (53.4)	20 (64.5)			51 (57.3)		
		Antipyretic (type, dose, and route)	Acetaminophen 650 mg PO	53 (91.4)			21 (67.7)			74 (83.1)	**0.006**	**0.073**
			Acetaminophen 100 mg PO	0 (0.0)			2 (6.5)			2 (2.2)		
			Acetaminophen 1 g PO	5 (8.6)			8 (25.8)			13 (14.6)		
Corticosteroid (type, dose, and route)			Hydrocortisone 100 mg IV	9 (15.5)	8 (25.8)	17 (19.1)	0.478			0.637		
			Hydrocortisone 100 mg IV + Prednisone 40 mg PO	1 (1.7)	0 (0.0)	1 (1.1)						
			Methylprednisolone 100 mg IV	41 (70.7)	18 (58.1)	59 (66.3)						
			Methylprednisolone 125 mg IV	4 (6.9)	1 (3.2)	5 (5.6)						
	Hydrocortisone 100 mg IV + Prednisone 5 mg PO		2 (3.4)	3 (9.7)	5 (5.6)							
	Methylprednisolone 62.5 mg IV		1 (1.7)	1 (3.2)	2 (2.2)							
	Corticosteroid acting class		Short acting	9 (15.5)	8 (25.8)	17 (19.1)		0.327	0.561			
		Short and intermediate acting	3 (5.2)	3 (9.7)	6 (6.7)					
		Intermediate acting	46 (79.3)	20 (64.5)	66 (74.2)					
		Antihistamine (type, dose, and route)	Diphenhydramine 50 mg IV	16 (27.6)	9 (29.0)	25 (28.1)					0.244	0.561
Cetirizine 10 mg PO			32 (55.2)	16 (51.6)	48 (53.9)							
Diphenhydramine 25 mg PO			7 (12.1)	1 (3.2)	8 (9.0)							
Diphenhydramine 50 mg IV + Loratadine 10 mg PO			1 (1.7)	3 (9.7)	4 (4.5)							
Loratadine 10 mg PO			0 (0.0)	1 (3.2)	1 (1.1)							
Diphenhydramine 25 mg IV			2 (3.4)	1 (3.2)	3 (3.4)							
Antihistamine generation			First and second generations	1 (1.7)	3 (9.7)	4 (4.5)	0.221			0.561		
			First-generation	25 (43.1)	11 (35.5)	36 (40.4)						
			Second-generation	32 (55.2)	17 (54.8)	49 (55.1)						
			Premedication strategies (detailed)	First-generation antihistamine + short-acting corticosteroid + acetaminophen	9 (15.5)	7 (22.6)							16 (18.0)	0.216	0.561
				First-generation antihistamine + short and intermediate acting corticosteroid + acetaminophen	2 (3.4)	0 (0.0)							2 (2.2)		
				First-generation antihistamine + intermediate-acting corticosteroid + acetaminophen	14 (24.1)	4 (12.9)							18 (20.2)		
				Second-generation antihistamine + intermediate-acting corticosteroid + acetaminophen	32 (55.2)	16 (51.6)							48 (53.9)		
				First and second generations antihistamine + short-acting corticosteroid + acetaminophen	0 (0.0)	1 (3.2)							1 (1.1)		

					Second-generation antihistamine + short and intermediate acting corticosteroid + acetaminophen	0 (0.0)							1 (3.2)			1 (1.1)		
	First and second generations antihistamine + short and intermediate acting corticosteroid + acetaminophen				1 (1.7)	2 (6.5)		3 (3.4)										

Categorical comparison was conducted using the Fisher Exact test.

Bold *p*-values are statistically significant (*p* < 0.05; *q* < 0.10).

### 3.5 Timing of infusion-related reactions (IRRs)

Among the 31 patients who experienced an IRR, 14 (45.2%) were classified as immediate IRRs, 16 (51.6%) as delayed IRRs, and 1 (3.2%) experienced both. When comparing baseline characteristics and premedication components between immediate and delayed reactions, no significant differences were found for age, sex, race, or MS phenotype. However, differences were observed between groups in certain premedication components. Corticosteroid type (*p* = 0.011), corticosteroid acting class (*p* = 0.016), antihistamine type (*p* = 0.018), antihistamine generation (*p* = 0.027), and overall premedication strategy (*p* = 0.040) showed statistically significant variation by reaction timing. All associations remained significant after FDR correction (*q* < 0.10). The differences between delayed and immediate reactions are presented in Supplementary Table 2.

### 3.6 Predictors of infusion-related reactions

A binomial logistic regression model was constructed to identify independent predictors of IRR occurrence. The overall model was not statistically significant (*p* = 0.332) and exhibited a predictive accuracy of 70.5%. After adjusting for MS type, DMT-naïve status, sex, and race, no individual premedication component or strategy emerged as a significant independent predictor of IRR (*p* > 0.05). The complete model details are presented in Supplementary Table 3.

## 4 Discussion

This study examined IRRs in 89 PwMS who initiated ublituximab therapy. We found a 34.8% IRR incidence. No baseline or premedication factors were significantly associated with IRR occurrence except for acetaminophen.

Our observed ublituximab IRR incidence (34.8%) was lower than the spectrum reported for most anti-CD20 therapies in MS in previous literature and somewhat lower than those from the original trial (47.7%) (5, 7, 8). In other clinical trials of anti-CD20 drugs, rituximab demonstrated higher IRR rates (67.1 to 78.3%), while ocrelizumab showed similar rates (34.3 to 39.9%) compared to ublituximab in our study (15, 16). Interestingly, subcutaneous ofatumumab exhibited lower systemic reactions (20.2%), in contrast to the high-dose intravenous formulations (78.9%), which suggests that the route of administration has some influence on the rate of IRRs besides its structure as a fully human antibody (16). Real-world studies reveal significantly lower IRR burdens compared to clinical trials. For example, rituximab-associated reactions occurred in only 7.8% of infusions in a Swedish cohort, and modified premedication in an Arab population reduced ocrelizumab IRRs to 26% (17, 18). The discordance between IRR rates reported in clinical trials and those observed in real-world practice is likely multifactorial, potentially driven by differences in patient selection, monitoring and reporting standards, infusion management approaches, and premedication strategies employed. While none of the premedication variables emerged as independent predictors of IRR occurrence, these findings should not be construed as evidence of inefficacy, as the study was neither designed nor powered to assess the effectiveness of specific premedication approaches.

Premedication composition influences anti-CD20 therapy IRR risk; previous studies identified corticosteroid selection and antihistamine generation as the main modulators (19–22). In a study addressing IRRs related to obinutuzumab, another glycoengineered drug, dexamethasone was associated with lower IRR rates than shorter-acting steroids (hydrocortisone or methylprednisolone) due to its longer half-life (36-54 h vs. 8-12 h) and the absence of hypersensitivity-inducing succinate esters. Consistent with these properties, longer-acting corticosteroids such as dexamethasone provide broader coverage across both early and delayed reactions (19, 20). Likewise, high-dose dexamethasone reduced first-infusion IRRs by 44% versus low-dose regimens (27.0% vs. 48.4%; OR = 6.49 for hydrocortisone-associated risk) (20). The relationship between corticosteroid pharmacokinetic profiles and the temporal distribution of IRRs remains undetermined, as the present dataset did not permit such an analysis. Regarding antihistamines, previous studies have reported lower IRR rates and reduced sedation with the second-generation compared with first-generation agents (20–22). Conversely, in our cohort, no significant differences were observed between antihistamine types in the occurrence of IRR. Acetaminophen dosing categories demonstrated a statistically significant association with IRRs in the univariate analyses (*p* = 0.006), which should be interpreted cautiously given the uneven distribution of patients among subgroups, with some dose categories containing insufficient numbers for robust comparison (specifically, the 100 mg dose category reflected a conservative clinical protocol at one site due to theoretical concerns of transaminitis, which had prompted the avoidance of acetaminophen during first infusions in the clinical trials). The observed association may reflect confounding practice patterns, or chance findings rather than a true biological effect. These observations highlight the need for prospective studies to evaluate whether specific antipyretic dosing strategies influence IRR outcomes during anti-CD20 therapy. Antipyretics were frequently used with other drugs in previous studies; nonetheless, there is no direct evidence of their efficacy in diminishing IRRs during anti-CD20 infusions (5, 20, 23). However, in the ULTIMATE trials, acetaminophen was not routinely given before the first dose and was only administered after pyrexia (if it occurred). This may partly explain the higher IRR rate observed. In our cohort, acetaminophen was administered prior to infusions. Antipyretics are thought to attenuate prostaglandin-mediated symptoms such as fever, headache, and malaise, which may have contributed to the lower frequency of mild IRRs observed, although this was not directly tested. Also, the analyses suggested variation in reaction timing among premedication categories, these results warrant cautious interpretation given the small number of patients within each subgroup, the heterogeneity of premedication protocols.

The glycoengineering of the Fc domain of ublituximab enhances FcγRIIIa affinity, amplifying antibody-dependent cellular cytotoxicity (ADCC) by 25-100-fold compared to other anti-CD20 agents (5, 7, 11). Despite theoretical advantages related to its mechanism of action, studies have reported higher IRR rates with ublituximab than with some other anti-CD20 therapies (5, 16). Proposed explanations include cytokine release associated with ADCC engagement and other immunologic factors (5, 24–26). Moreover, the antibody scaffold (chimeric vs. humanized) was initially implicated (5). The relative contributions of ADCC activity, antibody scaffold characteristics, and other factors to IRR risk remain incompletely characterized.

In our cohort, all observed IRRs were mild to moderate in severity, with no cases requiring hospitalization, treatment discontinuation, or grade ≥3 events. This contrasts with the pivotal ULTIMATE trials, where 2.8% of participants experienced grade 3 or higher reactions, including two grade 4 events, one anaphylaxis, and six discontinuations. The lower severity observed may relate to several factors, including optimized premedication and more cautious clinical monitoring. These findings reinforce the notion that IRRs with ublituximab, while common, are manageable in practice.

The strength of this study lies in its novelty as one of the first multicenter real-world analyses examining ublituximab-associated IRRs and premedication practices in patients with MS. The application of FDR correction adds robustness to the analyses; the observed associations persisted after adjustment for multiple comparisons. However, the findings should be interpreted with several important limitations in mind. First, the retrospective observational design limits causal inference and introduces the possibility of ascertainment bias, residual confounding, and chance findings. Potentially important confounders, including infusion-rate modifications, cumulative ublituximab exposure, center-specific infusion and premedication practices, prior allergic history, concomitant medications, body mass index, and atopic comorbidities, were not consistently available for inclusion in the model. Also, the modest sample size limits the ability to detect rare but clinically important adverse events. Data regarding healthcare utilization, and patient-reported burden were not systematically collected and therefore could not be evaluated. These outcomes may provide additional insight into the real-world clinical impact of IRRs and should be considered in future studies. In addition, premedication regimens were heterogeneous across participating centers, with many combinations represented by small subgroups. This heterogeneity reflects routine real-world practice but limits formal comparison between strategies and increases the risk of spurious associations. Subgroup comparisons should therefore be regarded as exploratory. Some corticosteroid doses used in this cohort were also lower than those commonly used in clinical trial protocols, which may have influenced IRR incidence and severity. Also, owing to sample size limitations, early and late post-infusion reactions were consolidated into a broader delayed IRR category in the analysis. This grouping, while pragmatic, may obscure clinically meaningful distinctions between reaction subtypes that could arise from different underlying mechanisms. Accordingly, this study should be considered descriptive and hypothesis-generating and warrant validation in larger cohorts. Future prospective studies using standardized premedication protocols and predefined IRR reporting measures will be important to better characterize factors associated with IRRs during ublituximab treatment.

## 5 Conclusion

In this real-world study of 89 MS patients initiating ublituximab, the overall IRR incidence was 34.8%, with both immediate and delayed reactions observed. No significant predictors of IRRs were identified. Considerable variability in premedication practices was observed across centers, reflecting current diversity in IRR mitigation approaches. Importantly, all IRRs observed in this study were mild to moderate in severity, suggesting that ublituximab infusions were generally well tolerated, which may relate to several factors, including the routine use of premedication and close clinical monitoring. Ublituximab’s glycoengineered design, while enhancing efficacy, potentiates cytokine-mediated IRRs, which may help explain the higher reaction rates compared to non-glycoengineered agents. Future prospective investigations incorporating standardized premedication protocols and detailed temporal and clinical characterization of IRRs are needed to inform strategies that optimize the benefit–risk profile of ublituximab.

## Data Availability

The raw data supporting the conclusions of this article will be made available by the corresponding author upon reasonable request, due to patient confidentiality restrictions.
