# Supplementary material for: Infusion-related reactions and premedication patterns in ublituximab-treated multiple sclerosis patients: a multicenter real-world study
Source: Front Neurol. 2026 Jul 16;17:1840196. doi: 10.3389/fneur.2026.1840196 (PMC13422192; doi:10.3389/fneur.2026.1840196)
Supplement: Supplementary file 1 [file Supplementary_Tables.docx]

**Supplementary table 1,** *Association between acetaminophen dose and IRR occurrence stratified by prior anti-CD20 exposure*

| Contingency Tables | | | | | | | | | |
| --- | --- | --- | --- | --- | --- | --- | --- | --- | --- |
|  | | | | **Infusion related reactions (IRR)** | | | |  | |
| **Anti-CD20 Naïve** | | **Antipyretic (type, dose, and route)** | | **No** | | **Yes** | | **Total** | |
| No |  | Acetaminophen 650 mg PO |  | 23 |  | 8 |  | 31 |  |
|  |  | Acetaminophen 100 mg PO |  | 0 |  | 1 |  | 1 |  |
|  |  | Acetaminophen 1g PO |  | 4 |  | 2 |  | 6 |  |
|  |  | Total |  | 27 |  | 11 |  | 38 |  |
| Yes |  | Acetaminophen 650 mg PO |  | 30 |  | 13 |  | 43 |  |
|  |  | Acetaminophen 100 mg PO |  | 0 |  | 1 |  | 1 |  |
|  |  | Acetaminophen 1g PO |  | 1 |  | 6 |  | 7 |  |
|  |  | Total |  | 31 |  | 20 |  | 51 |  |
| Total |  | Acetaminophen 650 mg PO |  | 53 |  | 21 |  | 74 |  |
|  |  | Acetaminophen 100 mg PO |  | 0 |  | 2 |  | 2 |  |
|  |  | Acetaminophen 1g PO |  | 5 |  | 8 |  | 13 |  |
|  |  | Total |  | 58 |  | 31 |  | 89 |  |
| \| χ² Tests \| \| \| \| \| \| \| \| \| --- \| --- \| --- \| --- \| --- \| --- \| --- \| --- \| \| **Anti-CD20 Naïve** \| \|  \| \| **Value** \| \| **p** \| \| \| No \|  \| Fisher's exact test \|  \|  \|  \| 0.296 \|  \| \|  \| \| N \|  \| 38 \|  \|  \| \| \| Yes \|  \| Fisher's exact test \|  \|  \|  \| **0.004** \|  \| \|  \| \| N \|  \| 51 \|  \|  \| \| \| Total \|  \| Fisher's exact test \|  \|  \|  \| **0.006** \|  \| \|  \| \| N \|  \| 89 \|  \|  \| \| \|  \| \| \| \| \| \| \| \| | | | | | | | | | |

**Supplementary table 2,** *Difference between different categories of baseline and premedication in terms of type of infusion-related reactions (IRR) [Delayed Vs. Immediate reaction]*

| Dependent: Type of Infusion related reactions (IRR) |  | Delayed IRR  16 (53.3) | Immediate IRR 14 (46.7) | Total | p value | q value |
| --- | --- | --- | --- | --- | --- | --- |
| MS type | RRMS | 13 (81.2) | 14 (100.0) | 27 (90.0) | 0.228 | 0.303 |
|  | SPMS | 3 (18.8) | 0 (0.0) | 3 (10.0) |  |  |
|  |  |  |  |  |  |  |
| Age | Median (IQR) | 45.0 (31.8 to 54.0) | 36.5 (29.0 to 44.8) | 39.0 (31.2 to 50.5) | 0.289 | 0.346 |
| Sex | Female | 9 (56.2) | 10 (71.4) | 19 (63.3) | 0.389 | 0.425 |
|  | Male | 7 (43.8) | 4 (28.6) | 11 (36.7) |  |  |
| Race | Black | 6 (40.0) | 4 (28.6) | 10 (34.5) | 0.700 | 0.700 |
|  | White | 9 (60.0) | 10 (71.4) | 19 (65.5) |  |  |
|  |  |  |  |  |  |  |
| DMT Naïve | No | 9 (56.2) | 12 (85.7) | 21 (70.0) | 0.118 | 0.203 |
|  | Yes | 7 (43.8) | 2 (14.3) | 9 (30.0) |  |  |
| Anti-CD20 Naïve | No | 4 (25.0) | 7 (50.0) | 11 (36.7) | 0.156 | 0.234 |
|  | Yes | 12 (75.0) | 7 (50.0) | 19 (63.3) |  |  |
| Antipyretic (type, dose, and route) | Acetaminophen 650 mg PO | 9 (56.2) | 12 (85.7) | 21 (70.0) | 0.103 | 0.203 |
|  | Acetaminophen 100 mg PO | 1 (6.2) | 1 (7.1) | 2 (6.7) |  |  |
|  | Acetaminophen 1g PO | 6 (37.5) | 1 (7.1) | 7 (23.3) |  |  |
| Corticosteroid (type, dose, and route) | Hydrocortisone 100 mg IV | 7 (43.8) | 1 (7.1) | 8 (26.7) | **0.011** | **0.070** |
|  | Methylprednisolone 100 mg IV | 6 (37.5) | 12 (85.7) | 18 (60.0) |  |  |
|  | Methylprednisolone 125 mg IV | 1 (6.2) | 0 (0.0) | 1 (3.3) |  |  |
|  | Hydrocortisone 100 mg IV + Prednisone 5 mg PO | 2 (12.5) | 0 (0.0) | 2 (6.7) |  |  |
|  | Methylprednisolone 62.5 mg IV | 0 (0.0) | 1 (7.1) | 1 (3.3) |  |  |
| Corticosteroid acting class | Short acting | 7 (43.8) | 1 (7.1) | 8 (26.7) | **0.016** | **0.070** |
|  | Short & Intermediate acting | 2 (12.5) | 0 (0.0) | 2 (6.7) |  |  |
|  | Intermediate acting | 7 (43.8) | 13 (92.9) | 20 (66.7) |  |  |
| Antihistamine (type, dose, and route) | Diphenhydramine 50 mg IV | 7 (43.8) | 2 (14.3) | 9 (30.0) | **0.018** | **0.070** |
|  | Cetirizine 10 mg PO | 5 (31.2) | 11 (78.6) | 16 (53.3) |  |  |
|  | Diphenhydramine 25 mg PO | 0 (0.0) | 1 (7.1) | 1 (3.3) |  |  |
|  | Diphenhydramine 50 mg IV + Loratadine 10 mg PO | 3 (18.8) | 0 (0.0) | 3 (10.0) |  |  |
|  | Diphenhydramine 25 mg IV | 1 (6.2) | 0 (0.0) | 1 (3.3) |  |  |
| Antihistamine generation | First & Second generations | 3 (18.8) | 0 (0.0) | 3 (10.0) | **0.027** | **0.070** |
|  | First-generation | 8 (50.0) | 3 (21.4) | 11 (36.7) |  |  |
|  | Second-generation | 5 (31.2) | 11 (78.6) | 16 (53.3) |  |  |
| Premedication strategies (detailed) | First-generation Antihistamine + Short-acting Corticosteroid + Acetaminophen | 6 (37.5) | 1 (7.1) | 7 (23.3) | **0.040** | **0.070** |
|  | First-generation Antihistamine + Intermediate-acting Corticosteroid + Acetaminophen | 2 (12.5) | 2 (14.3) | 4 (13.3) |  |  |
|  | Second-generation Antihistamine + Intermediate-acting Corticosteroid + Acetaminophen | 5 (31.2) | 11 (78.6) | 16 (53.3) |  |  |
|  | First & Second generations Antihistamine + Short-acting Corticosteroid + Acetaminophen | 1 (6.2) | 0 (0.0) | 1 (3.3) |  |  |
|  | First & Second generations Antihistamine + Short & Intermediate acting Corticosteroid + Acetaminophen | 2 (12.5) | 0 (0.0) | 2 (6.7) |  |  |

Categorical comparison was conducted using the Fisher Exact test.
Bold p-values are statistically significant (p<0.05, q<0.10).
One patient who experienced both reaction types was excluded, yielding n = 30. Percentages are calculated accordingly.

**Supplementary table 3, Binomial Logistic Regression tables,** *Prediction of IRR for different premedication strategies while controlling for confounding variables.* (Model Fit Measures, Omnibus Likelihood Ratio Tests, Model Coefficients, Collinearity Statistics, Classification Table, Predictive Measures (accuracy))

| **S table 3a, Model Fit Measures** | | | | | | | | | | | | | | | | |
| --- | --- | --- | --- | --- | --- | --- | --- | --- | --- | --- | --- | --- | --- | --- | --- | --- |
|  | | | | | | | | | | **Overall Model Test** | | | | | | |
| **Model** | | **Deviance** | | **AIC** | | **R²_McF_** | | **R²_CS_** | | **χ²** | | **df** | | | **p** | |
| 1 |  | 100 |  | 124 |  | 0.110 |  | 0.135 |  | 12.4 |  | 11 |  | | 0.332 |  |
| \| **S table 3b, Omnibus Likelihood Ratio Tests** \| \| \| \| \| --- \| --- \| --- \| --- \| \| Predictor \| χ² \| df \| p \| \| Sex \| 0.289 \| 1 \| .591 \| \| Race \| 2.244 \| 2 \| .326 \| \| MS type \| 1.021 \| 1 \| .312 \| \| Premedication strategies (detailed) \| 10.019 \| 6 \| .124 \| \| DMT Naïve \| 0.735 \| 1 \| .391 \|      \| **S table 3c, Assumption Checks; Collinearity Statistics** \| \| \| \| --- \| --- \| --- \| \|  \| VIF \| Tolerance \| \| Sex \| 1.06 \| 0.940 \| \| Race \| 1.06 \| 0.941 \| \| MS type \| 1.15 \| 0.866 \| \| Premedication strategies (detailed) \| 1.05 \| 0.955 \| \| DMT Naïve \| 1.10 \| 0.907 \| | | | | | | | | | | | | | |  |  |  |

| **S table 3d, Prediction: Classification Table** | | | |
| --- | --- | --- | --- |
|  | Predicted | |  |
| Observed | No | Yes | % Correct |
| No | 56 | 2 | 96.6 |
| Yes | 24 | 6 | 20.0 |
| Note. The cut-off value is set to 0.5 | | | |

| **S table 3e, Models coefficients** | | | | | | | |
| --- | --- | --- | --- | --- | --- | --- | --- |
|  | | | | | | **95% Confidence Interval** | |
| **Predictor** | **Estimate** | **SE** | **Z** | **p** | **Odds ratio** | **Lower** | **Upper** |
| Intercept | -0.300 | 0.674 | -0.44475 | .657 | 0.741 | 0.1979 | 2.78 |
| Sex: |  |  |  |  |  |  |  |
| Male – Female | -0.285 | 0.533 | -0.53508 | .593 | 0.752 | 0.2646 | 2.14 |
| Race: |  |  |  |  |  |  |  |
| White – Black | 0.619 | 0.578 | 1.07050 | .284 | 1.856 | 0.5981 | 5.76 |
| Asian – Black | -16.870 | 3956.180 | -0.00426 | .997 | 4.71e-8 | 0.0000 | Inf |
| MS type: |  |  |  |  |  |  |  |
| SPMS – RRMS | -0.826 | 0.848 | -0.97469 | .330 | 0.438 | 0.0831 | 2.31 |
| Premedication strategies (detailed): |  |  |  |  |  |  |  |
| First-generation Antihistamine + Short & Intermediate acting Corticosteroid + Acetaminophen – First-generation Antihistamine + Short-acting Corticosteroid + Acetaminophen | -17.309 | 2758.381 | -0.00628 | .995 | 3.04e-8 | 0.0000 | Inf |
| First-generation Antihistamine + Intermediate-acting Corticosteroid + Acetaminophen – First-generation Antihistamine + Short-acting Corticosteroid + Acetaminophen | -1.065 | 0.833 | -1.27787 | .201 | 0.345 | 0.0674 | 1.77 |
| Second-generation Antihistamine + Intermediate-acting Corticosteroid + Acetaminophen – First-generation Antihistamine + Short-acting Corticosteroid + Acetaminophen | -0.912 | 0.750 | -1.21597 | .224 | 0.402 | 0.0923 | 1.75 |
| First & Second generations Antihistamine + Short-acting Corticosteroid + Acetaminophen – First-generation Antihistamine + Short-acting Corticosteroid + Acetaminophen | 18.151 | 3956.180 | 0.00459 | .996 | 7.64e+7 | 0.0000 | Inf |
| Second-generation Antihistamine + Short & Intermediate acting Corticosteroid + Acetaminophen – First-generation Antihistamine + Short-acting Corticosteroid + Acetaminophen | 18.359 | 3956.180 | 0.00464 | .996 | 9.40e+7 | 0.0000 | Inf |
| First & Second generations Antihistamine + Short & Intermediate acting Corticosteroid + Acetaminophen – First-generation Antihistamine + Short-acting Corticosteroid + Acetaminophen | 1.009 | 1.472 | 0.68536 | .493 | 2.743 | 0.1531 | 49.13 |
| DMT Naïve: |  |  |  |  |  |  |  |
| Yes – No | 0.516 | 0.601 | 0.85892 | .390 | 1.675 | 0.5160 | 5.44 |
| Note. Estimates represent the log odds of "Infusion related reactions (IRR) = Yes" vs. "Infusion related reactions (IRR) = No" | | | | | | | |
